# Designing of peptides with desired half-life in intestine-like environment

**DOI:** 10.1186/1471-2105-15-282

**Published:** 2014-08-20

**Authors:** Arun Sharma, Deepak Singla, Mamoon Rashid, Gajendra Pal Singh Raghava

**Affiliations:** Bioinformatics Centre, Institute of Microbial Technology, Sector 39A, Chandigarh, India; Red Sea Research Center, King Abdullah University of Science and Technology, Thuwal, 23955-6900 Kingdom of Saudi Arabia

## Abstract

**Background:**

In past, a number of peptides have been reported to possess highly diverse properties ranging from cell penetrating, tumor homing, anticancer, anti-hypertensive, antiviral to antimicrobials. Owing to their excellent specificity, low-toxicity, rich chemical diversity and availability from natural sources, FDA has successfully approved a number of peptide-based drugs and several are in various stages of drug development. Though peptides are proven good drug candidates, their usage is still hindered mainly because of their high susceptibility towards proteases degradation. We have developed an *in silico* method to predict the half-life of peptides in intestine-like environment and to design better peptides having optimized physicochemical properties and half-life.

**Results:**

In this study, we have used 10mer (HL10) and 16mer (HL16) peptides dataset to develop prediction models for peptide half-life in intestine-like environment. First, SVM based models were developed on HL10 dataset which achieved maximum correlation R/R^2^ of 0.57/0.32, 0.68/0.46, and 0.69/0.47 using amino acid, dipeptide and tripeptide composition, respectively. Secondly, models developed on HL16 dataset showed maximum R/R^2^ of 0.91/0.82, 0.90/0.39, and 0.90/0.31 using amino acid, dipeptide and tripeptide composition, respectively. Furthermore, models that were developed on selected features, achieved a correlation (R) of 0.70 and 0.98 on HL10 and HL16 dataset, respectively. Preliminary analysis suggests the role of charged residue and amino acid size in peptide half-life/stability. Based on above models, we have developed a web server named HLP (**H**alf **L**ife **P**rediction), for predicting and designing peptides with desired half-life. The web server provides three facilities; i) half-life prediction, ii) physicochemical properties calculation and iii) designing mutant peptides.

**Conclusion:**

In summary, this study describes a web server ‘HLP’ that has been developed for assisting scientific community for predicting intestinal half-life of peptides and to design mutant peptides with better half-life and physicochemical properties. HLP models were trained using a dataset of peptides whose half-lives have been determined experimentally in crude intestinal proteases preparation. Thus, HLP server will help in designing peptides possessing the potential to be administered via oral route (http://www.imtech.res.in/raghava/hlp/).

**Electronic supplementary material:**

The online version of this article (doi:10.1186/1471-2105-15-282) contains supplementary material, which is available to authorized users.

## Background

Due to rapid advancement in peptide and peptidomimetic techniques, pharmaceutical companies are focusing towards peptides-based therapeutics [1]. A large number of peptides like insulin [2], cyclosporine [3], corticotropin [4] are used for treating various diseases like diabetes and immunoregulatory disorders [5–7]. Owing to their immense therapeutic importance, peptides have been curated from literature and stored in form of databases such as Hemolytik [8], PhytAMP [9], APD2 [10], DAMPD [11], CAMP [12], YADAMP [13], CPPsite [14], TumorHoPe [15], Quorumpeps [16], Brainpeps [17], MilkAMP [18], DADP [19], LAMP [20] and AVPdb [21] to name a few. Also, a number of computation tools such as CellPPD [22], TumorHPD [23], AntiCP [24], AVPpred [25] and ToxinPred [26] have been developed to predict and design cell penetrating, tumor homing, anticancer, antiviral and toxic peptides, respectively. Presently, most of the therapeutic peptides are used in the form of injection via subcutaneous, intravenous/intramuscular route. In addition, alternate routes such as pulmonary [27], oral [28, 29], intranasal [30], buccal [31], transdermal [32], ocular [33] and rectal [34] have been tested. Among all routes of drug delivery, oral is the most preferred route because oral formulations are less expensive and less prone to infection caused by inappropriate use/reuse of needles. Additionally, orally available peptides are highly accepted by patients, which increases the therapeutic value of the drug. However, designing and formulating an oral peptide has been considered as a challenging job. This is because of their undesirable physicochemical properties like large molecular size, high susceptibility to enzymatic degradation (proteases), hepatic and renal clearance, etc.

Amongst the above factors, peptide stability is one of the most difficult tasks to maintain. Their high susceptibility to proteases in the gut and serum (especially for cationic peptides) and fast degradation rate due to their arginine and lysine content makes them low orally bioavailable [5, 35, 36]. A major concern associated with the use of peptide therapeutics is improving their stability by protection against degrading proteases [37]. Although, a number of experimental techniques like peptide modification are available in order to improve peptide stability with high accuracy [5, 38], yet these experimental procedures are costly and time consuming. In view of its importance, there is a need to develop an *in silico* method for predicting 1) half-life of peptides as well as 2) prediction of mutations required in a peptide, in order to increase its intestinal half-life. In past, computational methods for predicting half-life of proteins/peptides in blood and kidney cells/cell lines have been developed. For example, ProtParam is a tool that helps to estimate half-life of a protein stored in Swiss-Prot/TrEMBL or a user entered protein. The estimation of half-life is done for three experimental models namely: yeast *in vivo*, mammalian reticulocytes (immature red blood cells) *in vitro* and Escherichia coli *in vivo* [39]. Stability Prediction tool predicts the stability of HIV-derived peptides in cytosolic extracts from human peripheral blood mononuclear cells [40]. SProtP is a web server to recognize the short-lived proteins (half-life < 30 minutes) in 293 T cells (a variant of human embryonic kidney cell line) [41, 42]. The N-end rule [43, 44] based ‘ProtLifePred server’ [http://protein-n-end-rule.leadhoster.com/] takes into account the ubiquitination (a process that involves post-translational modification of proteins) process of proteins [45–47] and predicts their stability in *S. cerevisiae*, *E. coli* and mammalian cells. Best of author’s knowledge there is no computational method developed for designing/predicting stability/half-life of peptides in intestine-like environment.

In order to facilitate researchers working in the field of therapeutic peptides, we have developed *in silico* models for predicting half-life of peptides. These models were developed using a set of peptides whose half-lives have been experimentally determined in crude intestinal proteases preparation [37]. Server developed in this study can be used to identify minimum mutations in a peptide required to optimize their half-life.

## Results

### Analysis of half-life data

**Figure 1:**
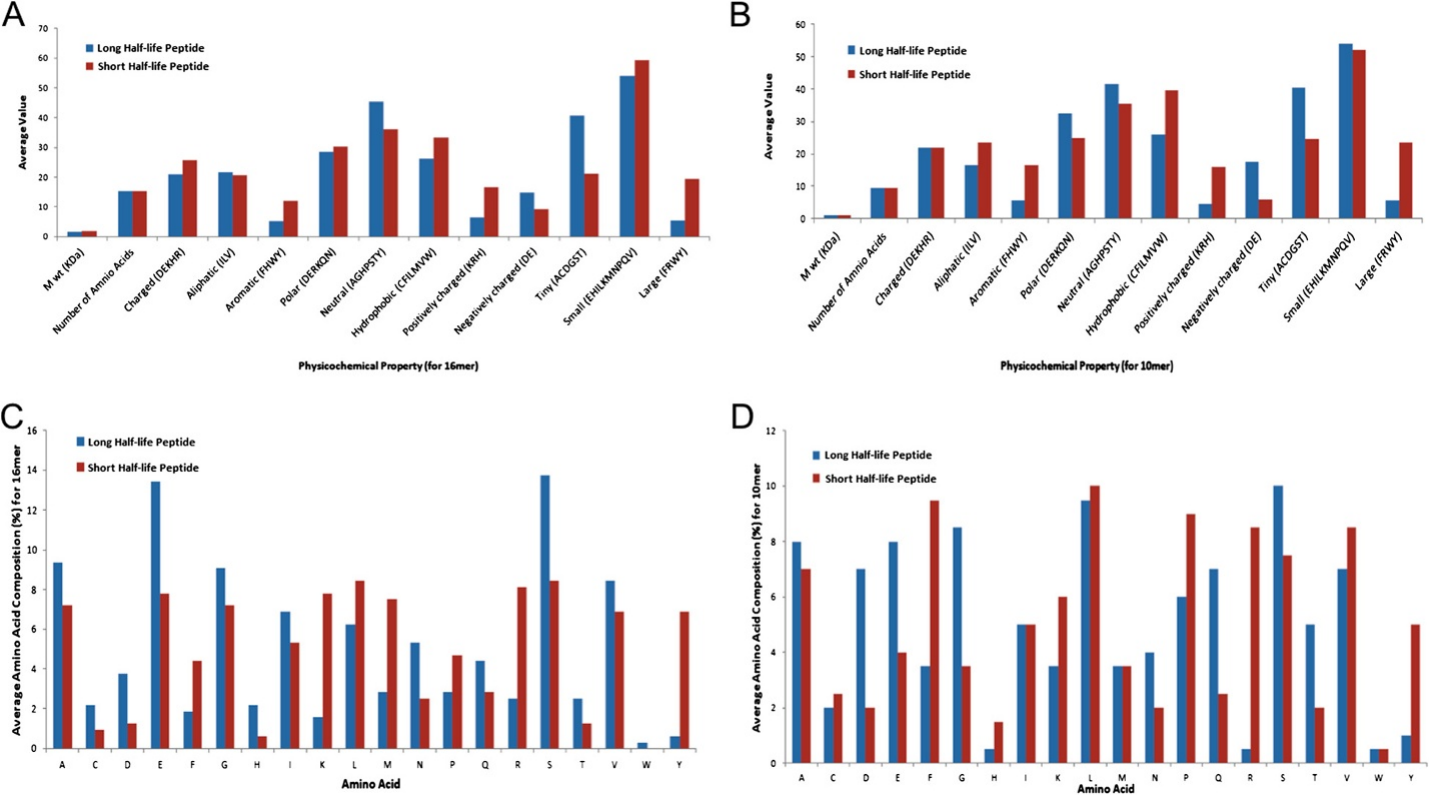
**Physicochemical properties and amino acid composition of top 20 peptides having longest half-life (stable peptides) and top 20 peptides having shortest half-life (unstable peptides). (A)** physicochemical properties of 16mer peptides having short and long half-life; **(B)** physicochemical properties of 10mer peptides having short and long half-life; **(C)** average amino acid composition 16mer peptides having short and long half-life; **(D)** shows average amino acid composition of 10mer peptides having short and long half-life.

We computed and analyzed physicochemical properties of peptides in both HL10 and HL16 datasets to understand relation between property of amino acids and their half-life. Based on the half-life value, peptides were classified into two categories peptides having long half-life (highly stable) and peptides having short half-life (poorly stable or unstable). Each category have top 20 peptides, it means 20 peptides having longest half-life were classified as stable and 20 peptides having shortest half-life were classified as unstable. We observed that negatively charged, neutral and tiny types of residues are more prominent in highly stable peptides (Figure 1A and B). We have also computed amino acid composition of peptides in highly and lowest/poorly stable peptide datasets. As shown in Figure 1 (C and D), residues Ala, Asp, Glu, Gly, Gln, Ser and Thr are abundant in peptide dataset with longer half-life. As evident from Additional file 1: Table S3, residues D (Asp), F (Phe), G (Gly), L (Leu), Q (Gln), R (Arg) and Y (Tyr) show significant differences (p < 0.05) in amino acid composition in 10mer dataset. Similarly, residues D (Asp), F (Phe), G (Gly), K (Lys), M (Met), N (Asn), R (Arg) and Y (Tyr) shows statistically significant differences (p < 0.05) in amino acid composition for 16mer dataset (Additional file 1: Table S4).

### Prediction of Half-Life

#### SVM based models on HL10 dataset

The model was developed on HL10 dataset using amino acid composition-based and achieved maximum R/R^2^ of 0.57/0.32 with mean absolute error (MAE) 1.87. Similarly models were developed using dipeptide and tripeptide composition resulted in R/R^2^ of 0.68/0.46 and 0.69/0.47 with MAE 1.44 and 1.38 respectively (Table 1). As shown in Table 1, performance of binary pattern based model is poor as compared to composition-based model with R/R^2^ of 0.22/0.02 and MAE 1.87. In order to understand role of residues at terminus, models were also developed using 5-residues from N and C terminal called 5 N-terminal and 5 C-terminal residues. We achieved maximum R/R^2^ of 0.39/0.12 and 0.32/0.09 using amino acid composition of 5 N-terminal and 5 C-terminal residues, respectively (Table 1). It is evident from the above results that composition-based models perform better than models based on binary profile/pattern.

**Table 1:** **The performance of SVM based models developed on HL10 dataset using different features of peptide sequences**

Input feature	Residues in peptides	Total attributes	R	R ^2^	MAE
**Amino acid composition**	All residues	20	0.57	0.32	1.87
	5 N-terminal		0.39	0.12	2.12
	5 C-terminal		0.32	0.09	1.99
**Binary pattern**	All residues	200	0.22	0.02	1.87
	5 N-terminal	100	0.06	-0.06	1.27
	5 C-terminal	100	0.34	0.12	2.01
**Dipeptide composition**	**All residues**	**400**	**0.68**	**0.46**	**1.44**
**Tripeptide composition**	All residues	8000	0.69	0.47	1.38

#### Models developed on selected features

After removing useless features, we developed models for predicting half-life using following techniques SMOreg, IBk, KStar and DecisionTable (implemented in Weka package). We developed composition-based models using best-selected features/compositions (four type of residues) and got maximum R/R^2^ of 0.61/0.35 with MAE 1.42 (Table 2, Additional file 1: Table S1). Similarly, we developed models using best-selected dipeptides (eight type of dipeptides) and achieved maximum R/R^2^ of 0.70/0.46 along with MAE 1.22. We also developed model using selected 38 tripeptides and obtained maximum R/R^2^ of 0.73/0.35 with MAE 1.39 (Table 2, Additional file 1: Table S1).

**Table 2:** **The performance of Weka models developed using selected features on HL10 dataset**

Total attributes	Techniques	Selected attributes	Method	R	R ^2^	MAE
20	KStar	D, G, P, R	Amino acid composition	0.61	0.35	1.42
**400**	**IBk**	**EK, EL, GD, GF, IE, KP, PG, YL**	**Dipeptide composition**	**0.70**	**0.46**	**1.22**
8000	IBk	AAH, AGR, AMP, ARE, ASV, DSI, EEK, ELY, ESK, FCI, FGD, FSL, FSS, FYC, GDS, GFG, GLF, GSI, GTS, ILP, INF, INK, IRN, ITK, KIL, KIS, KLP, LVL, MVL, PGF, PVQ, SGL, SIE, SLR, SVL, VFK, VLF, VYL	Tripeptide composition	0.73	0.35	1.39

### SVM based models on HL16 dataset

Similarly, SVM based models were developed on HL16 dataset. The amino acid composition-based model showed best performance with R/R^2^ of 0.91/0.82 with MAE 0.18 (Table 3). As shown in Table 3, the dipeptide and tripeptide composition based models showed an R/R^2^ of 0.90/0.39, and 0.90/0.31 with MAE 0.23 and 0.24, respectively. As compared to composition-based model, the binary pattern based model again showed poor performance with R/R^2^ of 0.13/0.01. Next, models were developed using 5 and 10 residues from N and C terminals, respectively. An increase in model performance with correlation value from 0.57 to 0.77 has been observed for N-terminal 5 to 10 residues based model developed using amino acid composition (Table 3). Similarly, the C-terminal based composition based model shows an increase in model performance with correlation (R) value of 0.37 to 0.81 and R^2^ of 0.13 to 0.65. The binary pattern based models performed poor than composition-based model; developed using N and C terminal residues (Table 3).

**Table 3:** **The performance SVM based models developed on HL16 dataset using composition and binary pattern**

Input features	Residues in peptides	Total attributes	R	R ^2^	MAE
**Amino acid composition**	**All residues**	**20**	**0.91**	**0.82**	**0.18**
	5 N-terminal		0.57	0.32	0.23
	10 N-terminal		0.77	0.60	0.24
	5 C-terminal		0.37	0.13	0.27
	10 C-terminal		0.81	0.65	0.24
**Binary pattern**	All residues	320	0.13	0.01	0.18
	5 N-terminal	100	0.17	0.02	0.20
	5 C-terminal	100	0.03	-0.002	0.18
	10 N-terminal	200	0.22	0.02	0.18
	10 C-terminal	200	0.09	-0.002	0.18
**Dipeptide composition**	All residues	400	0.90	0.39	0.23
**Tripeptide composition**	All residues	8000	0.90	0.31	0.24

### Models developed on selected features

We identified/selected best features (residues or dipeptides or tripeptides) having high correlation with half-life of peptides. The first model developed using four important features from the amino acid composition and achieved maximum R/R^2^ of 0.97/0.93 along with MAE 0.07 (Table 4, Additional file 1: Table S2). As shown in Table 4, the three selected dipeptide (CG, GD, GF) results in R/R^2^ value of 0.98/0.96 with MAE 0.06. Further model was developed on selected five attributes from tripeptide composition shows equal performance with correlation coefficient value of 0.98 (Table 4, Additional file 1: Table S2).

**Table 4:** **The performance of various models (Weka) using selected features on HL16 dataset**

Total attributes	Techniques	Selected attributes	Method	R	R ^2^	MAE
20	DecisionTable	C, D, G, R	Amino acid composition	0.97	0.93	0.07
**400**	**DecisionTable**	**CG, GD, GF**	**Dipeptide composition**	**0.98**	**0.96**	**0.06**
8000	DecisionTable	AQC, EAQ, FGD, GFG, QCG	Tripeptide composition	0.98	0.96	0.06

### Development of web server/software

Based on above study, we developed a web server for predicting intestinal half-life of peptides to design stable peptides through mutant generation and whole protein scanning. HLP server has three main modules called Peptide, Protein and Batch module. First module i.e., Peptide, allows users to submit single peptide at a time to the web server for predicting its intestinal half-life and designing mutants which may have better half-life and physicochemical properties than original peptide. Protein module allows a user to submit a protein for scanning to find peptide having high intestinal half-life. Furthermore, it allow to generate mutant peptides corresponding to user-selected peptide. Third module i.e., Batch, designed for high throughput scanning where a user can submit a large number of peptides for filtering best peptides (having high intestinal half-life and desired physicochemical properties). Our HLP server implement total six type of models; these can be divided in two classes: SVM based models (implemented using SVM^light^ package) and Weka based models (implemented using WEKA package). Our SVM based models implemented using following type of information of peptides; i) dipeptide composition in case of 10mer peptides, ii) amino acid composition for 16mer peptides and iii) type of composition depend on peptide length in case of user-selected sequence length. In case of Weka based models, following techniques and peptide information is used; i) IBK model using eight dipeptides (EK, EL, GD, GF, IE, KP, PG, YL) for 10mer peptides, ii) DecisionTable using three dipeptides (CG, GD, GF) for 16mer peptides and iii) model selection depends on peptide length in case of user-selected sequence length. The HLP web server was developed using Perl, CGI and HTML languages; it is available freely for public use from URL http://www.imtech.res.in/raghava/hlp/.

## Discussion

Although a number of peptides have entered in market against various indications (such as diabetes, hypertension, cancer, HIV, etc.) still one of the major challenges in success of peptide-based therapy is their low half-life in serum and gut. There are number of factors responsible for degrading or shortening half-life of peptides [5]; where *in silico* designing of peptides seems to be a promising field in the area of peptide based therapy. The presence of intestinal proteases is one of the major factors behind degradation of peptides. *In vitro* peptide stability screening is considered to be the most difficult and time-consuming task. In order to assist scientific community to screen stable peptide in intestine-like environment, we have made a systematic attempt in this direction. In this study, models have been developed for predicting half-life of peptides on dataset derived from Gorris *et al.*, 2009, where half-lives of peptides have been experimentally determined in the presence of crude intestinal proteases (high concentration). Firstly, they calculated half-life of peptides in undiluted proteolytic solution and then it has been subsequently extrapolated from results obtained from diluted solutions. The analysis based on these datasets suggest that charge and size of an amino acid are the two major factors governing the peptide stability. As reported in previous studies on bioactive peptides [22–24], positively charge amino acids Arg and Lys are preferred among majority of these kind of peptides while the present study reports these residues responsible to decrease peptide’s intestinal half-life. Therefore, the residues affecting peptide’s half-life and bioactivity should be optimized simultaneously while designing highly stable and potent bioactive peptides. The presence of large size amino acids such as Phe, Arg, Tyr and Trp will increase protease susceptibility and tiny amino acids (such as Gly, Ala, Ser, Thr) may help in their stabilization. Among these large-size amino acids, three (Phe, Tyr, Trp) belong to aromatic class and are also responsible for the decreased half-life. HLP models, developed on a small dataset shows good correlation (R) of 0.70 and 0.98 between predicted and actual half-life value on HL10 and HL16 dataset, respectively. One of two major limitations of our method is that it is not suitable for predicting the stability of modified peptides like N-terminal/C-terminal modification or effect of disulphide bond in a peptide. Therefore, our model is only suitable for predicting the stability of peptide composed of natural amino acids without any chemical modifications. Another limitation of the present study is the presence of small dataset in training and testing the half-life models due to unavailability of large dataset on peptide half-life in intestine from literature. The actual half-lives have been calculated in highly concentrated environment. Gorris *et al.* assume that the half-life values would be very high in real life. Unfortunately, they haven’t mentioned quantitatively, how high these half-lives values would be in real life. Thus, the half-life dataset helps in estimating half-lives of peptides relatively rather than in absolute terms. Just for our satisfaction and to test how good the models will work on peptides having very high half-lives, we developed new models after multiplying peptide’s actual half-life with 10 and 1000. As evident from Additional file 2: Tables S1-S6, half-life prediction models performs equally well for higher half-life containing peptides, irrespective of very high/low half-life. Hence, HLP can prove to be a extremely good resource to relatively estimate the half-life of peptides in intestine like environment. Additionally, this can help in improving half-life of peptide(s) half-life by mutating residue. We hope that our study will be useful to scientific community for designing/prediction of stable peptides and thus helpful in solving the major barrier in peptide based drug development.

## Conclusion

There is a growing demand of peptide-based drugs but the major bottleneck in their development is their short half-life. The present study can provide an efficient method to design therapeutic peptides having better half-life and physicochemical properties. We hope that our method would promote the usage of various types of therapeutic peptides in drug discovery and development.

## Methods

### Datasets

In this study, we have used two datasets called HL10 and HL16 for developing prediction models. The datasets containing peptides with their half-life, were obtained from Gorris *et al.*, 2009 [37]. HL10 dataset consists of 189 peptides with their half-life value varies from 0.0008 - 40.1296 seconds. HL16 dataset consists of 186 peptides with their half-life value varies from 0.0008 - 6.4211 seconds. Additionally, the distribution of half-lives (for both 10mer and 16mer) can be clearly seen from Figure five by Gorris *et al.*, 2009.

### Input features

#### Physicochemical properties

It is well established that activity or function of a peptide or protein depends on its amino acid sequence. Each amino acid has unique set of physicochemical properties like hydrophobicity, polarity, charge, size. Thus each peptide has different physicochemical properties depending on type of residue it contains. Thus, physicochemical properties of peptide play a significant role in determining the activity of peptides; therefore we had calculated more than 15 different properties of peptides like hydrophobicity, volume, isoelectric point, flexibility [48–51].

#### Residue composition as input features

In the past, it has been observed that amino acids composition encapsulates the protein information. Based on this approach, numbers of methods have been developed like protein folding rate prediction, sub-cellular localization prediction [51, 52]. In this study, we computed amino acid, dipeptide and tripeptide composition and used as input feature for developing prediction models. These compositions are represented by a vector of dimension 20, 400, and 8000 for residue/amino acid, dipeptide and tripeptide composition respectively [53].

#### Binary pattern

Each amino acid of a peptide is represented by binary pattern of 20, where 1′s represents the presence of concerned amino acid at that position and 0′s for the absence of other 19 amino acids (e.g. Ala is represented by 1,0,0,0,0,0,0,0,0,0,0,0,0,0,0,0,0,0,0,0) [22]. Thus, a vector of dimension *N X* 20 is used to represent the peptide of length *N (size of peptide)*.

### Models based on machine learning techniques

In this study various machine-learning techniques have been used for developing regression models. In order to implement support vector machine for developing models we used commonly used highly efficient software called SVM^light^. This package allows to implement various kernels (e.g., linear, polynomial, RBF) as well as it allow to tune various parameters.

In addition to SVM^light^, we also used Weka, a Java based software package having various machine learning and feature selection algorithms [54]. In this study, we have used SMOreg (Sequential Minimization Optimization) module of Weka for building model for predicting half-life/stability of peptides. We also used IBK, KStar and DecisionTable based classifier, implemented in Weka for model building and optimization.

### Feature selection

Previously, studies have shown that all input features are not equally important during the construction of an *in silico* model [55]. Thus, feature selection seems to be more relevant in order to find out the most important and contributing input features. In this study for feature selection, we have used CfsSubsetEval attribute evaluator with BestFirst (with parameters: -D 1 -N 5) search method (using full training set as attribute selection mode) implemented in Weka software for both HL10 and HL16 dataset.

### Evaluation of performance

The performance of the models was evaluated by employing a five-fold cross-validation technique. The whole dataset is divided into five sets in such a way that every time four sets are used for training and one set for testing. This process is repeated five times in such a way that each set is used for testing. Once the model is constructed, fitness has been accessed using the following statistical parameters.


Where y_i_ and x_i_ represent predicted and actual half-life values for i_th_ peptide. N is total number of peptides. SD is the sum of the squared deviations between the activities of the test set and mean activities of the training peptides. The value of correlation coefficient (R) and coefficient of determination (R^2^) is used to measure the quality of model. The value of R varies from -1 to +1 while R^2^ is from 0 to 1. The negative value of R shows the negative correlation with a particular property or feature. Thus, higher the value of R and R^2^, better will be the quality of model in term of prediction of half-life of peptides.

### Availability and requirements

We have developed a web server HLP, freely available for predicting half-life of peptides in intestine-like environment. This web server was developed using PERL, CGI and HTML programming languages.

## Electronic supplementary material

Additional file 1: **Weka based results for both HL10 (Table S1) and HL16 (Table S2) using selected features.** Differences in amino acid composition for longest half-life (stable) Vs shortest half-life (unstable) containing 10mer **(Table S3)** and 16mer peptides **(Table S4)**. (XLS 35 KB)

Additional file 2: **Performance of SVM and Weka based models (Tables S1-S6) after multiplying their actual half-life with 10 and 1000.** Longest half-life (Stable) and shortest half-life containing 10mer **(Table S7)** and 16mer peptides **(Table S8)**. (DOC 419 KB)
