# Supplementary material for: Designing of peptides with desired half-life in intestine-like environment
Source: BMC Bioinformatics. 2014 Aug 20;15(1):282. doi: 10.1186/1471-2105-15-282 (PMC4150950; doi:10.1186/1471-2105-15-282)
Supplement: Supplementary file 2 — Additional file 2: Performance of SVM and Weka based models (Tables S1-S6) after multiplying their actual half-life with 10 and 1000. Longest half-life (Stable) and shortest half-life containing 10mer (Table S7) and 16mer peptides (Table S8). (DOC 419 KB) [file 12859_2014_6549_MOESM2_ESM.doc]

**Table S1. Performance of SVM based models developed using composition and binary pattern of peptides on HL10 dataset.**

| **Input Feature** | **Residues in**  **Peptides** | **Total**  **Attributes** | **Evaluation  Parameters** | **Actual Half-life** | **Actual Half-life*10** | **log(Actual Half-life*10)** | **Actual Half-life*1000** | **log(Actual Half-life*1000)** |
| --- | --- | --- | --- | --- | --- | --- | --- | --- |
| **Amino Acid**  **Composition** | All Residues | 20 | **R** | 0.57 | 0.48 | 0.805 | 0.22 | 0.805 |
|  |  |  | **R^2^** | 0.32 | 0.17 | 0.65 | -0.07 | 0.65 |
|  |  |  | **MAE** | 1.87 | 15.34 | 1.09 | 1244.05 | 1.09 |
|  | 5 N-terminal |  | **R** | 0.39 | 0.32 | 0.48 | 0.09 | 0.48 |
|  |  |  | **R^2^** | 0.12 | 0.05 | 0.23 | -0.07 | 0.23 |
|  |  |  | **MAE** | 2.12 | 15.12 | 1.68 | 1254.36 | 1.68 |
|  | 5 C-terminal |  | **R** | 0.32 | 0.26 | 0.52 | 0.26 | 0.52 |
|  |  |  | **R^2^** | 0.09 | -0.06 | 0.27 | -0.07 | 0.27 |
|  |  |  | **MAE** | 1.99 | 12.49 | 1.63 | 1251.81 | 1.63 |
| **Binary Pattern** | All residues | 200 | **R** | 0.22 | 0.17 | 0.38 | 0.18 | 0.38 |
|  |  |  | **R^2^** | 0.02 | -0.07 | 0.12 | -0.07 | 0.12 |
|  |  |  | **MAE** | 1.87 | 12.50 | 1.71 | 1254.11 | 1.71 |
|  | 5 N-terminal | 100 | **R** | 0.06 | 0.12 | 0.22 | 0.16 | 0.22 |
|  |  |  | **R^2^** | -0.06 | -0.07 | 0.01 | -0.07 | 0.01 |
|  |  |  | **MAE** | 1.27 | 12.54 | 1.85 | 1254.22 | 1.85 |
|  | 5 C-terminal | 100 | **R** | 0.34 | 0.21 | 0.33 | 0.14 | 0.33 |
|  |  |  | **R^2^** | 0.12 | -0.05 | -0.01 | -0.07 | -0.01 |
|  |  |  | **MAE** | 2.01 | 12.93 | 1.96 | 1254.41 | 1.96 |
| **Dipeptide Composition** | **All residues** | **400** | **R** | **0.68** | **0.62** | **0.86** | **0.36** | **0.86** |
|  |  |  | **R^2^** | **0.46** | **0.19** | **0.72** | **-0.06** | **0.72** |
|  |  |  | **MAE** | **1.44** | **11.77** | **0.95** | **1257.21** | **0.95** |
| **Tripeptide Composition** | All residues | 8000 | **R** | 0.69 | 0.62 | 0.86 | 0.33 | 0.86 |
|  |  |  | **R^2^** | 0.47 | 0.21 | 0.69 | -0.07 | 0.69 |
|  |  |  | **MAE** | 1.38 | 12.22 | 1.02 | 1248.66 | 1.02 |

**Table S2. Performance of models developed using selected features on HL10 dataset.**

| **Total attributes** | **Techniques** | **Selected attributes** | **Method** | **Evaluation  Parameters** | **Actual Half-life** | **Actual Half-life*10** | **log(Actual Half-life*10)** | **Actual Half-life*1000** | **log(Actual Half-life*1000)** |
| --- | --- | --- | --- | --- | --- | --- | --- | --- | --- |
| 20 | KStar | D, G, P, R | Amino Acid Composition | **R** | 0.61 | 0.61 | 0.53 | 0.61 | 0.53 |
|  |  |  |  | **R^2^** | 0.35 | 0.35 | 0.26 | 0.35 | 0.26 |
|  |  |  |  | **MAE** | 1.42 | 14.23 | 1.63 | 1423.18 | 1.63 |
| **400** | **IBk** | **EK, EL, GD, GF, IE, KP, PG, YL** | **Dipeptide Composition** | **R** | **0.70** | **0.70** | **0.38** | **0.70** | **0.38** |
|  |  |  |  | **R^2^** | **0.46** | **0.46** | **0.14** | **0.46** | **0.14** |
|  |  |  |  | **MAE** | **1.22** | **12.24** | **1.76** | **1224.13** | **1.76** |
| 8000 | IBk | AAH, AGR, AMP, ARE, ASV, DSI, EEK, ELY, ESK, FCI, FGD, FSL, FSS, FYC, GDS, GFG, GLF, GSI, GTS, ILP, INF, INK, IRN, ITK, KIL, KIS, KLP, LVL, MVL, PGF, PVQ, SGL, SIE, SLR, SVL, VFK, VLF, VYL | Tripeptide Composition | **R** | 0.73 | 0.73 | 0.47 | 0.73 | 0.47 |
|  |  |  |  | **R^2^** | 0.35 | 0.35 | 0.17 | 0.35 | 0.17 |
|  |  |  |  | **MAE** | 1.39 | 13.93 | 1.75 | 1392.96 | 1.75 |

**Table S3. Performance of the composition and binary pattern based SVM models developed on HL16 dataset.**

| **Input Features** | **Residues in Peptides** | **Total Attributes** | **Evaluation  Parameters** | **Actual Half-life** | **Actual Half-life*10** | **log(Actual Half-life*10)** | **Actual Half-life*1000** | **log(Actual Half-life*1000)** |
| --- | --- | --- | --- | --- | --- | --- | --- | --- |
| **Amino Acid**  **Composition** | All residues | 20 | **R** | **0.91** | 0.89 | 0.88 | **0.29** | **0.88** |
|  |  |  | **R^2^** | **0.82** | 0.47 | 0.77 | **-0.003** | **0.77** |
|  |  |  | **MAE** | **0.18** | 1.48 | 0.59 | **140.65** | **0.59** |
|  | 5 N-terminal |  | **R** | 0.57 | 0.57 | 0.59 | 0.16 | 0.59 |
|  |  |  | **R^2^** | 0.32 | 0.16 | 0.32 | -0.006 | 0.32 |
|  |  |  | **MAE** | 0.23 | 1.57 | 1.04 | 143.97 | 1.04 |
|  | 10 N-terminal |  | **R** | 0.77 | 0.73 | 0.88 | 0.32 | 0.88 |
|  |  |  | **R^2^** | 0.60 | 0.28 | 0.77 | 0.02 | 0.77 |
|  |  |  | **MAE** | 0.24 | 1.34 | 0.60 | 137.8 | 0.60 |
|  | 5 C-terminal |  | **R** | 0.37 | 0.34 | 0.54 | 0.22 | 0.54 |
|  |  |  | **R^2^** | 0.13 | 0.09 | 0.29 | 0 | 0.29 |
|  |  |  | **MAE** | 0.27 | 1.78 | 1.04 | 149.38 | 1.04 |
|  | 10 C-terminal |  | **R** | 0.81 | 0.71 | 0.85 | 0.35 | 0.85 |
|  |  |  | **R^2^** | 0.65 | 0.32 | 0.73 | 0.02 | 0.73 |
|  |  |  | **MAE** | 0.24 | 1.55 | 0.65 | 135.91 | 0.65 |
| **Binary Pattern** | All residues | 320 | **R** | 0.13 | 0.20 | 0.28 | 0.14 | 0.28 |
|  |  |  | **R^2^** | 0.01 | -0.01 | -0.24 | -0.02 | -0.19 |
|  |  |  | **MAE** | 0.18 | 1.37 | 1.47 | 139.09 | 1.43 |
|  | 5 N-terminal | 100 | **R** | 0.17 | 0.15 | 0.12 | 0.06 | 0.12 |
|  |  |  | **R^2^** | 0.02 | 0.01 | -0.24 | -0.03 | -0.24 |
|  |  |  | **MAE** | 0.20 | 1.99 | 1.45 | 132.76 | 1.45 |
|  | 5 C-terminal | 100 | **R** | 0.03 | 0.09 | 0.12 | 0.09 | 0.12 |
|  |  |  | **R^2^** | -0.002 | -0.002 | -0.49 | -0.01 | -0.49 |
|  |  |  | **MAE** | 0.18 | 1.57 | 1.62 | 152.29 | 1.62 |
|  | 10 N-terminal | 200 | **R** | 0.22 | 0.19 | 0.27 | 0.15 | 0.27 |
|  |  |  | **R^2^** | 0.02 | 0.02 | -0.08 | -0.02 | -0.08 |
|  |  |  | **MAE** | 0.18 | 1.68 | 1.35 | 136.5 | 1.35 |
|  | 10 C-terminal | 200 | **R** | 0.09 | 0.23 | 0.19 | 0.16 | 0.19 |
|  |  |  | **R^2^** | -0.002 | -0.008 | -0.22 | -0.02 | -0.22 |
|  |  |  | **MAE** | 0.18 | 1.39 | 1.46 | 137.27 | 1.46 |
| **Dipeptide Composition** | All residues | 400 | **R** | 0.90 | 0.89 | **0.92** | **0.45** | **0.92** |
|  |  |  | **R^2^** | 0.39 | 0.40 | **0.84** | 0.03 | **0.84** |
|  |  |  | **MAE** | 0.23 | 1.59 | **0.46** | 127.4 | **0.46** |
| **Tripeptide Composition** | All residues | 8000 | **R** | 0.90 | **0.88** | **0.92** | **0.48** | **0.92** |
|  |  |  | **R^2^** | 0.31 | 0.31 | **0.84** | 0.03 | **0.84** |
|  |  |  | **MAE** | 0.24 | 1.71 | **0.45** | 129 | **0.45** |

**Table S4. Performance of composition based models on selected features developed on HL16 dataset.**

| **Total attributes** | **Techniques** | **Selected attributes** | **Method** | **Evaluation  Parameters** | **Actual Half-life** | **Actual Half-life*10** | **log(Actual Half-life*10)** | **Actual Half-life*1000** | **log(Actual Half-life*1000)** |
| --- | --- | --- | --- | --- | --- | --- | --- | --- | --- |
| 20 | DecisionTable | C, D, G, R | Amino Acid Composition | **R** | 0.97 | 0.96 | 0.60 | 0.96 | 0.60 |
|  |  |  |  | **R^2^** | 0.93 | 0.93 | 0.35 | 0.93 | 0.35 |
|  |  |  |  | **MAE** | 0.07 | 0.66 | 1.05 | 66.28 | 1.05 |
| **400** | **DecisionTable** | **CG, GD, GF** | **Dipeptide Composition** | **R** | **0.98** | **0.98** | **0.48** | **0.98** | **0.48** |
|  |  |  |  | **R^2^** | **0.96** | **0.96** | **0.23** | **0.96** | **0.23** |
|  |  |  |  | **MAE** | **0.06** | **0.58** | **1.20** | **57.96** | **1.20** |
| 8000 | **DecisionTable** | AQC, EAQ, FGD, GFG, QCG | Tripeptide Composition | **R** | 0.98 | 0.98 | 0.51 | 0.98 | 0.51 |
|  |  |  |  | **R^2^** | 0.96 | 0.96 | 0.26 | 0.96 | 0.26 |
|  |  |  |  | **MAE** | 0.06 | 0.58 | 1.19 | 57.94 | 1.19 |

**Table S5. Results of HL10 dataset on selected features**

| **Input Feature** | **Total attributes** | **Selected attributes** | **Techniques** | **Parameter** | **Evaluation  Parameters** | **Actual Half-life** | **Actual Half-life*10** | **log(Actual Half-life*10)** | **Actual Half-life*1000** | **log(Actual Half-life*1000)** |
| --- | --- | --- | --- | --- | --- | --- | --- | --- | --- | --- |
| Amino Acid composition | 20 | D, G, P, R | IBk | -K 1 -W 0 | **R** | 0.59 | 0.59 | 0.55 | 0.59 | 0.55 |
|  |  |  |  |  | **R^2^** | 0.24 | 0.24 | 0.29 | 0.24 | 0.29 |
|  |  |  |  |  | **MAE** | 1.58 | 15.77 | 1.55 | 1577.26 | 1.55 |
|  |  |  | **KStar** | **-B 20 -M a** | **R** | **0.61** | **0.61** | **0.53** | **0.61** | **0.53** |
|  |  |  |  |  | **R^2^** | **0.35** | **0.35** | **0.26** | **0.35** | **0.26** |
|  |  |  |  |  | **MAE** | **1.42** | **14.23** | **1.63** | **1423.18** | **1.63** |
|  |  |  | DecisionTable | -X 1 -S | **R** | 0.33 | 0.33 | 0.50 | 0.33 | 0.50 |
|  |  |  |  |  | **R^2^** | 0.02 | 0.02 | 0.25 | 0.02 | 0.25 |
|  |  |  |  |  | **MAE** | 1.60 | 15.97 | 1.62 | 1597.38 | 1.62 |
|  |  |  | SMOreg Puk | -C 1.0 -N 0 | **R** | 0.57 | 0.57 | 0.55 | 0.57 | 0.55 |
|  |  |  |  |  | **R^2^** | 0.31 | 0.31 | 0.28 | 0.31 | 0.28 |
|  |  |  |  |  | **MAE** | 1.28 | 12.78 | 1.57 | 1278.91 | 1.56 |
|  |  |  | SMOreg RBF | -C 1.0 -N 0 | **R** | 0.35 | 0.36 | 0.45 | 0.35 | 0.45 |
|  |  |  |  |  | **R^2^** | -0.06 | -0.06 | 0.14 | -0.06 | 0.14 |
|  |  |  |  |  | **MAE** | 1.25 | 12.49 | 1.75 | 1250.66 | 1.75 |
| Dipeptide composition | 400 | **EK, EL, GD, GF, IE, KP, PG, YL** | **IBk** | **-K 3 -W 0** | **R** | **0.70** | **0.70** | **0.38** | 0.70 | **0.38** |
|  |  |  |  |  | **R^2^** | **0.46** | **0.46** | **0.14** | 0.46 | **0.14** |
|  |  |  |  |  | **MAE** | **1.22** | **12.24** | **1.76** | 1224.13 | **1.76** |
|  |  |  | KStar | -B 20 -M a | **R** | 0.66 | 0.66 | 0.42 | **0.66** | 0.42 |
|  |  |  |  |  | **R^2^** | 0.41 | 0.41 | 0.17 | **0.41** | 0.17 |
|  |  |  |  |  | **MAE** | 1.32 | 13.08 | 1.74 | **1318.12** | 1.74 |
|  |  |  | DecisionTable | -X 1 -S | **R** | 0.48 | 0.48 | 0.32 | 0.48 | 0.32 |
|  |  |  |  |  | **R^2^** | 0.11 | 0.11 | 0.09 | 0.11 | 0.09 |
|  |  |  |  |  | **MAE** | 1.62 | 16.22 | 1.82 | 1621.72 | 1.82 |
|  |  |  | SMOreg Puk | -C 3.0 -N 0 | **R** | 0.57 | 0.57 | 0.42 | 0.57 | 0.42 |
|  |  |  |  |  | **R^2^** | 0.26 | 0.26 | 0.16 | 0.26 | 0.16 |
|  |  |  |  |  | **MAE** | 1.24 | 12.43 | 1.73 | 1246.11 | 1.73 |
|  |  |  | SMOreg RBF | -C 1.0 -N 0 | **R** | 0.62 | 0.62 | 0.37 | 0.62 | 0.37 |
|  |  |  |  |  | **R^2^** | 0.12 | 0.12 | 0.11 | 0.12 | 0.11 |
|  |  |  |  |  | **MAE** | 1.19 | 11.91 | 1.80 | 1190.87 | 1.80 |
| Tripeptide composition | 8000 | AAH, AGR, AMP, ARE, ASV, DSI, EEK, ELY, ESK, FCI, FGD, FSL, FSS, FYC, GDS, GFG, GLF, GSI, GTS, ILP, INF, INK, IRN, ITK, KIL, KIS, KLP, LVL, MVL, PGF, PVQ, SGL, SIE, SLR, SVL, VFK, VLF, VYL | **IBk** | **-K 4 -W 0** | **R** | **0.73** | **0.73** | **0.47** | **0.73** | **0.47** |
|  |  |  |  |  | **R^2^** | **0.35** | **0.35** | **0.17** | **0.35** | **0.17** |
|  |  |  |  |  | **MAE** | **1.39** | **13.93** | **1.75** | **1392.96** | **1.75** |
|  |  |  | KStar | -B 90 -M a | **R** | 0.62 | 0.62 | 0.44 | 0.62 | 0.44 |
|  |  |  |  |  | **R^2^** | 0.33 | 0.33 | 0.18 | 0.33 | 0.18 |
|  |  |  |  |  | **MAE** | 1.53 | 15.26 | 1.73 | 1525.64 | 1.73 |
|  |  |  | DecisionTable | -X 1 -S | **R** | 0.65 | 0.65 | 0.65 | 0.65 | 0.65 |
|  |  |  |  |  | **R^2^** | 0.36 | 0.36 | 0.41 | 0.36 | 0.41 |
|  |  |  |  |  | **MAE** | 1.32 | 13.19 | 1.34 | 1319.29 | 1.34 |
|  |  |  | SMOreg Puk | -C 1.0 -N 0 | **R** | 0.54 | 0.54 | 0.70 | 0.54 | 0.70 |
|  |  |  |  |  | **R^2^** | 0.27 | 0.27 | 0.48 | 0.26 | 0.48 |
|  |  |  |  |  | **MAE** | 1.16 | 11.62 | 1.25 | 1159.51 | 1.25 |
|  |  |  | SMOreg RBF | -C 1.0 -N 0 | **R** | 0.71 | 0.71 | 0.68 | 0.71 | 0.67 |
|  |  |  |  |  | **R^2^** | 0.22 | 0.22 | 0.26 | 0.22 | 0.26 |
|  |  |  |  |  | **MAE** | 1.13 | 11.27 | 1.59 | 1126.40 | 1.59 |

**Table S6. Results of HL16 dataset on selected features**

| **Input Feature** | **Total attributes** | **Selected attributes** | **Techniques** | **Parameter** | **Evaluation  Parameters** | **Actual Half-life** | **Actual Half-life*10** | **log(Actual Half-life*10)** | **Actual Half-life*1000** | **log(Actual Half-life*1000)** |
| --- | --- | --- | --- | --- | --- | --- | --- | --- | --- | --- |
| Amino Acid composition | 20 | **C, D, G, R** | IBk | -K 3 -W 0 | **R** | 0.98 | 0.98 | 0.51 | 0.98 | 0.51 |
|  |  |  |  |  | **R^2^** | 0.77 | 0.77 | 0.26 | 0.77 | 0.26 |
|  |  |  |  |  | **MAE** | 0.09 | 0.92 | 1.10 | 91.48 | 1.10 |
|  |  |  | KStar | -B 3 -M a | **R** | 0.96 | 0.96 | 0.53 | 0.96 | 0.55 |
|  |  |  |  |  | **R^2^** | 0.91 | 0.91 | 0.26 | 0.91 | 0.29 |
|  |  |  |  |  | **MAE** | 0.07 | 0.68 | 1.04 | 68.27 | 1.08 |
|  |  |  | **DecisionTable** | **-X 1 -S** | **R** | **0.97** | **0.96** | **0.60** | **0.96** | **0.60** |
|  |  |  |  |  | **R^2^** | **0.93** | **0.93** | **0.35** | **0.93** | **0.35** |
|  |  |  |  |  | **MAE** | **0.07** | **0.66** | **1.05** | **66.28** | **1.05** |
|  |  |  | SMOreg Puk | -C 1.0 -N 0 | **R** | 0.90 | 0.90 | 0.46 | 0.90 | 0.46 |
|  |  |  |  |  | **R^2^** | 0.79 | 0.79 | 0.13 | 0.79 | 0.13 |
|  |  |  |  |  | **MAE** | 0.09 | 0.90 | 1.10 | 89.66 | 1.10 |
|  |  |  | SMOreg RBF | -C 20.0 -N 0 | **R** | 0.44 | 0.20 | 0.42 | 0.24 | 0.42 |
|  |  |  |  |  | **R^2^** | -0.01 | -0.02 | 0.14 | -0.02 | 0.14 |
|  |  |  |  |  | **MAE** | 0.13 | 1.31 | 1.16 | 131.53 | 1.16 |
| Dipeptide composition | 400 | CG, GD, GF | IBk | -K 7 -W 0 | **R** | 0.97 | 0.98 | 0.43 | 0.98 | 0.43 |
|  |  |  |  |  | **R^2^** | 0.35 | 0.35 | 0.12 | 0.35 | 0.12 |
|  |  |  |  |  | **MAE** | 0.12 | 1.18 | 1.28 | 118.47 | 1.28 |
|  |  |  | KStar | -B 20 -M a | **R** | 0.90 | 0.90 | 0.46 | 0.90 | 0.46 |
|  |  |  |  |  | **R^2^** | 0.82 | 0.82 | 0.22 | 0.82 | 0.22 |
|  |  |  |  |  | **MAE** | 0.09 | 0.88 | 1.22 | 87.73 | 1.22 |
|  |  |  | **DecisionTable** | **-X 1 -S** | **R** | **0.98** | **0.98** | **0.48** | **0.98** | **0.48** |
|  |  |  |  |  | **R^2^** | **0.96** | **0.96** | **0.23** | **0.96** | **0.23** |
|  |  |  |  |  | **MAE** | **0.06** | **0.58** | **1.20** | **57.96** | **1.20** |
|  |  |  | SMOreg Puk | -C 5.0 -N 0 | **R** | 0.93 | 0.92 | 0.45 | 0.92 | 0.45 |
|  |  |  |  |  | **R^2^** | 0.85 | 0.85 | 0.18 | 0.85 | 0.18 |
|  |  |  |  |  | **MAE** | 0.08 | 0.83 | 1.25 | 82.88 | 1.25 |
|  |  |  | SMOreg RBF | -C 1.0 -N 0 | **R** | 0.25 | 0.33 | 0.29 | 0.33 | 0.29 |
|  |  |  |  |  | **R^2^** | 0.05 | 0.06 | 0.07 | 0.06 | 0.07 |
|  |  |  |  |  | **MAE** | 0.16 | 1.39 | 1.30 | 139.27 | 1.30 |
| Tripeptide composition | 8000 | AQC, EAQ, FGD, GFG, QCG | IBk | -K 7 -W 0 | **R** | 0.98 | 0.98 | 0.44 | 0.98 | 0.44 |
|  |  |  |  |  | **R^2^** | 0.43 | 0.43 | 0.14 | 0.43 | 0.14 |
|  |  |  |  |  | **MAE** | 0.11 | 1.12 | 1.26 | 111.46 | 1.26 |
|  |  |  | KStar | -B 15 -M a | **R** | 0.93 | 0.93 | 0.53 | 0.93 | 0.53 |
|  |  |  |  |  | **R^2^** | 0.85 | 0.85 | 0.28 | 0.85 | 0.28 |
|  |  |  |  |  | **MAE** | 0.08 | 0.76 | 1.16 | 75.52 | 1.16 |
|  |  |  | **DecisionTable** | **-X 1 -S** | **R** | **0.98** | **0.98** | **0.51** | **0.98** | **0.51** |
|  |  |  |  |  | **R^2^** | **0.96** | **0.96** | **0.26** | **0.96** | **0.26** |
|  |  |  |  |  | **MAE** | **0.06** | **0.58** | **1.19** | **57.94** | **1.19** |
|  |  |  | SMOreg Puk | -C 2.0 -N 0 | **R** | 0.94 | 0.94 | 0.46 | 0.94 | 0.46 |
|  |  |  |  |  | **R^2^** | 0.89 | 0.89 | 0.20 | 0.89 | 0.20 |
|  |  |  |  |  | **MAE** | 0.08 | 0.79 | 1.24 | 78.86 | 1.24 |
|  |  |  | SMOreg RBF | -C 2.0 -N 0 | **R** | 0.34 | 0.35 | 0.43 | 0.35 | 0.43 |
|  |  |  |  |  | **R^2^** | 0.10 | 0.10 | 0.18 | 0.10 | 0.18 |
|  |  |  |  |  | **MAE** | 0.15 | 1.46 | 1.26 | 146.23 | 1.26 |

**Table S7. Peptide sequence and half-life for long-half life and short half-life peptides from HL10 dataset**

| **Long Half-life Peptides** | | | **Short Half-life Peptides** | | |
| --- | --- | --- | --- | --- | --- |
| **Sr. No.** | **Peptide sequence (10mer)** | **Half-life (in seconds)** | **Sr. No.** | **Peptide sequence (10mer)** | **Half-life (in seconds)** |
| **1** | **KLPGFGDSIE** | **40.1296** | **1** | **PFLFCIKHIA** | **0.0046** |
| **2** | **FDKLPGFGDS** | **25.2445** | **2** | **KVYLPRMKME** | **0.0044** |
| **3** | **LNQITKPNDV** | **20.6999** | **3** | **DHPFLFCIKH** | **0.0042** |
| **4** | **TEQESKPVQM** | **19.4782** | **4** | **VFKGLWEKAF** | **0.0039** |
| **5** | **PGFGDSIEAQ** | **17.8114** | **5** | **GIIRNVLQPS** | **0.0038** |
| **6** | **DSIEAQCGTS** | **13.4815** | **6** | **MEERKIKVYL** | **0.0037** |
| **7** | **AEERYPILPE** | **11.4998** | **7** | **DVYSFSLASR** | **0.0037** |
| **8** | **VLLPDEVSGL** | **8.3587** | **8** | **FSLASRLYAE** | **0.0035** |
| **9** | **AFKDEDTQAM** | **7.6573** | **9** | **EDTQAMPFRV** | **0.0033** |
| **10** | **FGDSIEAQCG** | **7.1220** | **10** | **LASRLYAEER** | **0.0031** |
| **11** | **IEAQCGTSVN** | **6.4814** | **11** | **TRTQINKVVR** | **0.0027** |
| **12** | **PSSVDSQTAM** | **4.5647** | **12** | **PNDVYSFSLA** | **0.0026** |
| **13** | **AQCGTSVNVH** | **4.3435** | **13** | **MYQIGLFRVA** | **0.0025** |
| **14** | **DILNQITKPN** | **4.1593** | **14** | **YSFSLASRLY** | **0.0019** |
| **15** | **VVGSAEAGVD** | **4.0618** | **15** | **KIKVYLPRMK** | **0.0017** |
| **16** | **PFASGTMSML** | **3.8440** | **16** | **TNAVLFFGRC** | **0.0015** |
| **17** | **QESKPVQMMY** | **3.8079** | **17** | **QIGLFRVASM** | **0.0015** |
| **18** | **INSWVESQTN** | **3.3348** | **18** | **VLFFGRCVSP** | **0.0014** |
| **19** | **GSAEAGVDAA** | **3.1271** | **19** | **AVLFFGRCVS** | **0.0012** |
| **20** | **GSIGAASMEF** | **2.7013** | **20** | **ERKIKVYLPR** | **0.0008** |

**Table S8. Peptide sequence and half-life for long-half life and short half-life peptides from HL16 dataset**

| **Long Half-life Peptides** | | | **Short Half-life Peptides** | | |
| --- | --- | --- | --- | --- | --- |
| **Sr. No.** | **Peptide sequence (16mer)** | **Half-life (in seconds)** | **Sr. No.** | **Peptide sequence (16mer)** | **Half-life (in seconds)** |
| **1** | **KLPGFGDSIEAQCGTS** | **6.4211** | **1** | **SRLYAEERYPILPEYL** | **0.0028** |
| **2** | **FDKLPGFGDSIEAQCG** | **5.6930** | **2** | **FRVASMASEKMKILEL** | **0.0026** |
| **3** | **PGFGDSIEAQCGTSVN** | **5.0348** | **3** | **QITKPNDVYSFSLASR** | **0.0025** |
| **4** | **FGDSIEAQCGTSVNVH** | **1.3951** | **4** | **MYQIGLFRVASMASEK** | **0.0025** |
| **5** | **DSIEAQCGTSVNVHSS** | **0.4688** | **5** | **CIKHIATNAVLFFGRC** | **0.0025** |
| **6** | **SSSANLSGISSAESLK** | **0.3682** | **6** | **LASRLYAEERYPILPE** | **0.0023** |
| **7** | **AEERYPILPEYLQCVK** | **0.2682** | **7** | **PVQMMYQIGLFRVASM** | **0.0022** |
| **8** | **VFSSSANLSGISSAES** | **0.2471** | **8** | **SSNVMEERKIKVYLPR** | **0.0021** |
| **9** | **VLLPDEVSGLEQLESI** | **0.2344** | **9** | **QMMYQIGLFRVASMAS** | **0.0021** |
| **10** | **SSLRDILNQITKPNDV** | **0.1963** | **10** | **QIGLFRVASMASEKMK** | **0.0021** |
| **11** | **EINEAGREVVGSAEAG** | **0.1950** | **11** | **NVMEERKIKVYLPRMK** | **0.0021** |
| **12** | **IEAQCGTSVNVHSSLR** | **0.1930** | **12** | **SKPVQMMYQIGLFRVA** | **0.0020** |
| **13** | **ISQAVHAAHAEINEAG** | **0.1789** | **13** | **KIKVYLPRMKMEEKYN** | **0.0016** |
| **14** | **NEAGREVVGSAEAGVD** | **0.1676** | **14** | **PNDVYSFSLASRLYAE** | **0.0014** |
| **15** | **NVLQPSSVDSQTAMVL** | **0.1618** | **15** | **MEERKIKVYLPRMKME** | **0.0014** |
| **16** | **LPDEVSGLEQLESIIN** | **0.1597** | **16** | **ERKIKVYLPRMKMEEK** | **0.0012** |
| **17** | **HAEINEAGREVVGSAE** | **0.1531** | **17** | **TKPNDVYSFSLASRLY** | **0.0011** |
| **18** | **ELINSWVESQTNGIIR** | **0.1455** | **18** | **DVYSFSLASRLYAEER** | **0.0011** |
| **19** | **AAHAEINEAGREVVGS** | **0.1269** | **19** | **HIATNAVLFFGRCVSP** | **0.0009** |
| **20** | **AGREVVGSAEAGVDAA** | **0.1212** | **20** | **KHIATNAVLFFGRCVS** | **0.0008** |
